# Comparison of the Chondrogenic Differentiation Potential of Equine Synovial Membrane-Derived and Bone Marrow-Derived Mesenchymal Stem Cells

**DOI:** 10.3389/fvets.2019.00178

**Published:** 2019-06-06

**Authors:** Alexis L. Gale, Renata L. Linardi, George McClung, Renata M. Mammone, Kyla F. Ortved

**Affiliations:** ^1^Department of Clinical Studies, New Bolton Center, School of Veterinary Medicine, University of Pennsylvania, Philadelphia, PA, United States; ^2^VCA San Francisco Veterinary Specialists, San Francisco, CA, United States

**Keywords:** mesenchymal stem cells, synovial membrane, bone marrow, equine, cartilage repair, chondrogenesis

## Abstract

Focal cartilage injury occurs commonly and often precipitates OA. Mesenchymal stem cells (MSCs) may be useful for repairing cartilage lesions, thereby preventing joint degeneration. Although MSCs isolated from bone marrow have been shown to have chondrogenic potential, synovial membrane-derived MSCs (SM-MSCs) may have superior chondrogenic abilities due to a common progenitor cell between synovium and cartilage. The objective of this study was to directly compare the immunophenotype, proliferative capabilities, and chondrogenic potential of equine SM-MSCs and bone marrow-derived MSCs (BM-MSCs). In order to do this, MSCs were isolated from synovial membrane and bone marrow collected from 6 adult horses. Flow cytometric analysis was used to assess cell surface marker expression including CD29, CD44, CD90, CD105, CD45, CD-79α, MHCI, and MHCII. Proliferation rates and doubling time were quantified in P1 and P2 cells. Trilineage differentiation assays were performed. MSC pellets were cultured in chondrogenic induction media for 28 days. Pellets were stained with toluidine blue to assess proteoglycan deposition. Expression of the chondrogenic-related genes *ACAN, COL2b*, and *SOX9* was quantified using qRT-PCR. The immunophenotypes of BM-MSCs and SM-MSCs were similar with both cell types being positive for expression of stem cell markers (CD29, CD44, CD90, CD105, and MHCI) and negative for exclusion markers (CD45 and CD79α). Although SM-MSCs did not express the exclusion marker, MHCII, expression of MHCII was moderate in BM-MSCs. Overall, chondrogenic differentiation was not significantly between the cell types with histologic parameters, proteoglycan content and gene expression being similar. BM-MSCs showed enhanced osteogenic differentiation compared to SM-MSCs. Synovial membrane is a feasible source of MSCs in the horse, however, superior chondrogenesis *in vitro* should not be expected under currently described culture conditions.

## Introduction

Trauma to articular cartilage occurs commonly and often leads to focal chondral defects. Due to the poor intrinsic healing capabilities of cartilage, full thickness defects are repaired with biomechanically inferior fibrocartilage which can lead to global degeneration of the joint or post-traumatic osteoarthritis (PTOA) (1, 2). Resurfacing of chondral defects could restore the articular surface and help prevent the development of PTOA. Currently, there are no effective disease-modifying OA drugs to halt or reverse OA, therefore, preventing the development of PTOA remains paramount. Cell-based cartilage repair strategies have been intensely investigated, with many techniques being used clinically.

Autologous chondrocyte implantation (ACI) has been one of the most commonly employed techniques for the repair of large cartilaginous defects in man (3, 4). Both autologous and allogeneic chondrocyte implantation have been described with some success in the horse (5, 6). Despite improved clinical outcomes and healing, ACI has several limitations including the need for multiple surgical procedures, graft hypertrophy (7), and donor site morbidity (8). Additionally, immune responses to allogeneic chondrocytes have limited its applicability. Considering the limitations of chondrocyte implantation, an alternative cell source for resurfacing the articular surface would be beneficial.

Mesenchymal stem cells (MSCs) represent a potentially useful source for cartilage repair as they are easily accessible, can be expanded in the laboratory, and are multipotent (9). To date, the vast majority of cell-based cartilage repair has been focused on bone marrow-derived MSCs (BM-MSCs) (10–12). Bone marrow can be obtained from the sternum or ilium of the standing horse and then expanded in culture for future use (13). Chondrogenic differentiation can be promoted by adding TGF-β1 or TGF-β3 to culture medium (14, 15), however, studies evaluating long-term repair of full thickness chondral defects in the horse have been disappointing (11). Recently, synovial-derived MSCs (SM-MSCs) have been proposed as an alternative source of MSCs due to potential superior chondrogenic capabilities.

Synovial membrane-derived MSCs (SM-MSCs) have been purified from synovium in humans (16), rats (17) and the horse (18). SM-MSC transplantation into chondral defects in a rabbit model showed improved *in vivo* healing over other types of MSCs (19). Improved chondrogenesis of SM-MSCs may be explained in part by the presence of a common progenitor cell between synovium and cartilage, and that SM-MSCs have higher CD44 (hyaluronan receptor) expression and can express uridine disphosphoglucose dehydrogenase (UDPGD), an enzyme needed for hyaluronan synthesis (20). Although SM-MSCs have been shown to have superior chondrogenic potential compared to BM-MSCs in other species, a direct comparison between these two cell sources in the horse is lacking (21, 22). Currently, the majority of studies evaluating chondrogenesis of equine synovial membrane- or synovial fluid-MSCs do not directly compare these cell sources to BM-MSCs (18, 23–26). If equine SM-MSCs have superior chondrogenic capabilities compared to BM-MSCs, synovial membrane should be considered as a source of progenitor cells for treatment of chondral lesions. Synovium can be harvested in the standing horse or during arthroscopic procedures, and SM-MSCs can be isolated and expanded in the lab in preparation for chondrogenic differentiation. Since the horse is the most relevant large animal model for human cartilage repair, comparing chondrogenesis of SM-MSCs to BM-MSCs is vital to enhancing cell-based repair techniques.

The main objective of this study was to directly compare the immunophenotype, and proliferative and chondrogenic capabilities of equine SM-MSCs and BM-MSCs in order to evaluate SM-MSCs as a source for cartilage repair. We hypothesized that SM-MSCs would have a similar immunophenotype to BM-MSCs but would have superior proliferative and chondrogenic capabilities.

## Methods

### Animals

Six, systemically healthy horses between the ages of 2–6 years were used in the study. This study was carried out in accordance with the recommendations of the Institutional Animal Care and Use Committee (IACUC) at the University of Pennsylvania.

### Bone Marrow Collection and Culture

Bone marrow was collected aseptically from the sternebrae of horses being euthanized for unrelated reasons immediately following euthanasia. Using an 11-gauge Jamshidi bone marrow biopsy needle (VWR Scientific, Bridgeport, NJ) and 60 mL syringe containing 30,000 U of heparin, 30 mL of bone marrow was aspirated. Bone marrow samples were processed via density centrifugation with Ficoll-Paque Plus (GE Healthcare, Chicago, IL, USA) prior to seeding into flasks containing medium consisting of Dulbecco's Modified Eagle Medium (DMEM) with 1 g/L of D-glucose, 2 mM L-glutamine, and 1 mM sodium pyruvate (ThermoFisher Scientific, Hampton, NH), penicillin (100 U/mL)-streptomycin (100 μg/mL) solution (Invitrogen, Carlsbad, CA), 10% fetal bovine serum (FBS) (VWR Life Science Seradigm, VWR, Radnor, PA), and basic fibroblastic growth factor (bFGF, 1 ng/mL) (Invitrogen, Carlsbad, CA). Medium was changed every 48 h. Cells were passaged when they reached ~80% confluency using Trypsin-EDTA Cell Dissociation Reagent (ThermoFisher Scientific, Waltham, MA). Passage 2 (P2) cells were used for differentiation assays. Cell number and viability was determined using the Cellometer Auto 2000 Cell Viability Counter (Nexcelom Bioscience, Lawrence, MA) and ViaStain™ AOPI staining solution (Nexcelom Bioscience LLC, Lawrence, MA).

### Synovial Membrane Collection and Culture

Synovial membrane (SM) was collected from the same horses immediately following bone marrow aspiration. All synovial membrane was collected aseptically from dorsal aspect of the antebrachiocarpal and middle carpal joint of normal carpi. Following harvest, synovial membrane was rinsed in phosphate buffered saline (saline) with penicillin (100 U/mL) and streptomycin (100 μg/mL). Synovial membrane (~400 mg) was then debrided with a sterile syringe plunger and incubated at 37°C in 200 μL FBS for 20 min. Samples were re-suspended in DMEM with 4.5 g/L D-glucose, 2 mM L-Glutamine, and 1 mM sodium pyruvate (ThermoFisher Scientific, Hampton, NH), penicillin (100 U/mL)-streptomycin (100 μg/mL) solution, and 10% FBS. Medium was changed every 48 h. Cells were passaged when they reached ~ 80% confluency using Trypsin-EDTA Cell Dissociation Reagent (Gibco™, ThermoFisher Scientific, Waltham, MA). Passage 2 cells were used for differentiation assays. Cell number and viability was determined using the Cellometer™ Auto 2000 Cell Viability Counter and ViaStain™ AOPI staining solution.

### Proliferation Assay

Passage 1 and 2 cells were seeded into 6-well tissue culture plates at a density of 3,000 cells/cm^2^. At 24, 48, 72, and 96 h, cells were detached using 0.25% trypsin-EDTA dissociation reagent. Cell number and viability was determined using the Cellometer™ Auto 2000 Cell Viability Counter and ViaStain™ AOPI staining solution. Calculation of doubling time (DT) at 96 h was calculated using the following formula:

$$\begin{array}{l} \text{DT}=\text{t}x\log2/\left(\log\text{N}\text{t}/\log\text{N}0\right) \end{array}$$

Where *t* is the incubation time in hours, *N*t is the number of cells at the end of the incubation time, and *N*0 is the number of cells at time = 0. All assays were performed in triplicate.

### Immunophenotyping

Flow cytometric analysis using specific markers for stemness was performed on P2 cells in order to evaluate the immunophenotype of the different cell populations. Prior to flow cytometry, cells were collected using Accutase® Cell Detachment Solution (Innovative Cell Technologies, Inc., San Diego, CA) in order to preserve cell surface markers (27). Cells (1 x 10^5^) were placed in 96-well round bottom plates and washed twice with PBS. Cell pellets were resuspended in 100 μL of PBS with 0.5% bovine serum albumin (BSA) (Sigma Aldrich, St. Louis, MO) and 0.02% sodium azide (Thermo Fisher scientific, Waltham, MA) and incubated at 4°C for 20 min. Cells were then incubated with 50 μL of the appropriate primary antibody at 4°C for 45 min, rinsed twice with PBS, and then resuspended in the secondary antibody (50 μL) when appropriate and incubate at 4°C for 45 min. After the final PBS rinse, the pellets were re-suspended in 200 μL of PBS containing 7-AAD (7-Aminoactinomycin D, Thermo Fisher scientific, Waltham, MA). Cells were stained with anti-CD29, CD44, CD90, CD105, CD45, CD-79α, MHCI, and MHCII antibodies and isotype controls were used to establish fluorescent gates. Table 1 shows the antibodies and isotype controls used. Flow cytometry and subsequent analysis was performed using a Guava® easyCyte 8HT Benchtop Flow Cytometer (Millipore Sigma, Burlington, MA).

**Table 1:** Antibodies used for flow cytometric analysis of equine cell surface markers.

**Antibody**	**Clone/Isotype**	**Host species**	**Target species**	**Fluorophore**	**2^**°**^ Antibody**	**Company**	**Dilution for 1^**°**^ antibody**
CD29	TDM29/IgG1^a^	Mouse	Human	APC	Yes Goat anti-mouse IgG	EMD Millipore	1:100
CD44	IM7/IgG2b^b^	Rat	Human	FITC	No	Thermo IM7	1:80
CD90	?/IgM	Mouse	Canine, Equine	RPE	No	WSU Monoclonal Antibody Center	1:200
CD105	SN6/IgG1^b^	Mouse	Human	Alexa 488	No	Bio Rad	1:10
CD45RB	?/IgM	Mouse	Equine	RPE	No	WSU Monoclonal Antibody Center	1:200
CD79α	HM57/IgG1^c^	Mouse	Human	Alexa 647	No	Bio Rad	1:200
MHCI	cz3/IgG2b	Mouse	Equine	APC	Yes Goat anti-mouse IgG	Gift^d^	1:100
MHCII	cz11/IgG1	Mouse	Equine	APC	Yes Goat anti-mouse IgG	Gift^d^	1:200
**Isotype control**	**Corresponding** **MAB**		**Target species**	**Fluorophore**		**Company**	**Dilution**
IgG1	To CD29		Mouse	APC		Abcam	1:100
IgG2b	To CD44		Rat	Alexa 488		Abcam	1:100
IgM	To CD90		Mouse	PE		Abcam	1:200
IgG1	To CD105		Mouse	Alexa 488		Abcam	1:200
IgM	To CD45RB		Mouse	PE		Abcam	1:200
IgG1	To CD79α		Mouse	Alexa 647		Abcam	1:400
IgG2b	To MHCI		Mouse	APC		Abcam	1:100
IgG1	To MHCII		Mouse	APC		Abcam	1:100

a *Validated by Laval et al. (28)*.

b *Validated by Paebst et al. (29)*.

c *Validated by De Schauwer et al. (30)*.

d *Gifts from Dr. Doug Antczak, Cornell University, Ithaca, New York, USA*.

### Trilineage Differentiation Assay

Osteogenic, adipogenic, and chondrogenic differentiation assays were performed using P2 SM-MSCs and BM-MSCs. For osteogenic differentiation, cells were seeded into 6-well culture plates in SM-MSC or BM-MSC medium at a seeding density of 2,900 cells/cm^2^. After 48 h, osteogenic differentiation medium was added containing basal differentiation medium consisting of Advanced DMEM/F12, 1% sodium pyruvate (Gibco Life Technologies, Carlsbad, CA), 25 mM HEPES buffer, 4 mM L-glutamine (Thermo Fisher Scientific, Waltham, MA), and penicillin (100 U/mL)-streptomycin (100 μg/mL) solution. The basal medium was supplemented with β-glycerophosphate (2.2 μg/mL) (Sigma Aldrich, St. Louis, MO), dexamethasone (8 μg/mL) (Sigma-Aldrich, St.Louis, MO), 2-phospho-L-ascorbic acid (0.05 mg/mL) (Sigma-Aldrich, St. Louis, MO), and 10% FBS. Cells are cultured in osteogenic medium for 14 days. Media was changed every 48 h. For each horse, control SM-MSCs and BM-MSCs were maintained in basal medium appropriate to the cell type for the duration of the culture. Following 14 days of culture, cells were rinsed with PBS and fixed with 10% formalin before staining with 2% alizarin red (Sigma-Aldrich, St. Louis, MO) at pH 4.2 for confirmation of extra-cellular calcium matrix.

For adipogenic differentiation, cells were seeded into 6-well tissue culture plates containing basal medium at a density of 5,100 cells/ cm^2^. After 48 h, the medium in the treatment wells was changed to adipogenic induction medium consisting of the basal differentiation medium outlined above supplemented with biotin (8 μg/mL) (Sigma-Aldrich, St. Louis, MO), calcium pantothenate (4 μg/mL) (Sigma-Aldrich, St. Louis, MO), insulin (5.8 μg/mL) (Sigma-Aldrich, Stl Louis, MO), dexamethasone (4 μg/mL), isobutylmethylxanthine (0.1 mg/mL) (Sigma-Aldrich, St. Louis, MO), rosiglithizone (0.0178 mg/mL) (Sigma-aldrich, St. Louis, MO), 5% rabbit serum (Thermo Fisher Scientific, Waltham, MA), and 3% FBS. Medium was changed every 48 h. After 6 days in induction medium, the medium was changed to adipogenic maintenance medium using the same reagents without rosiglithisone or isobutylmethylxanthine. For each horse, control SM-MSCs and BM-MSCs were maintained in the cell-type specific basal medium for the duration of the culture. Following 14 days of culture, cells were rinsed with PBS and fixed with 10% formalin before staining with Oil Red O (Sigma-Aldrich Corp., St. Louis, MO) for confirmation of lipid droplet accumulation in the cytoplasm of cells.

For chondrogenic differentiation, 500,000 cells were pelleted in 15 mL conical tubes via centrifugation at 400 g for 10 min. After 48 h in the appropriate basal medium for the cell type, chondrogenesis was induced with chondrogenic media containing of DMEM, 4.5 g/L D-glucose, 1% sodium pyruvate, L-Glutamine (4 mM), HEPES buffer (25 mM), and penicillin (100 U/mL)-streptomycin (100 μg/mL) supplemented with TGF-β3 (0.01 μg/mL) (Thermo Fisher Scientific, Waltham, MA), dexamethasone (0.4 μg/mL), 2- phospho-L-ascorbic acid (0.05 μg/mL), proline (0.04 mg/mL) (Thermo Fisher Scientific, Waltham, MA), 1% insulin-transferrin-selenium solution (Thermo Fisher Scientific, Waltham, MA), and 1% FBS. Pellets were maintained in culture for 28 days. For each horse, control SM-MSCs and BM-MSCs were maintained in basal medium for the duration of the culture. At the end of the culture period, pellets were fixed in a 10% formalin solution prior to paraffin embedding and sectioning. Pellets were then stained with hematoxylin and eosin (H&E) and toluidine blue.

### Gene Expression

For osteogenic and adipogenic differentiation, RNA was isolated using the Qiagen RNeasy Mini Kit (Qiagen, Germantown, MD). For chondrogenic differentiation, pellets were biopulverized in liquid nitrogen using a multiple sample stainless steel biopulverizer and hammer (BioSpec Products, Inc., Bartlesville, OK). The Qiagen RNeasy Fibrous Tissue Mini Kit (Qiagen, Germantown, MD) was then used to complete RNA isolation. For all samples, RNA concentration and purity were quantified using a UV microspectrophotometer (NanoDrop™ One, ThermoFisher Scientific, Waltham, MA). Complementary DNA was prepared using a High Capacity cDNA Reverse Transcription kit (ThermoFisher Scientific, Waltham, MA) and an Eppendorf master cycler (Hamburg, Germany). Real-time quantitative PCR was performed using TaqMan™ Master mix and the Applied Biosystems™ QuantStudio™ 6 Flex Real-Time PCR System (Applied Biosystem, Foster City, CA). Primers and probes were designed using NCBI Primer-BLAST and Integrated DNA Technologies (IDT) PrimerQuest Tool software and synthesized by IDT (Coralville, IA) (Table 2). The following genes were analyzed: *PPAR*γ for adipogenesis; runt-related transcription factor-2 (*RUNX2*), alkaline phosphate (*ALP*), and osteocalcin (*OC*) for osteogenesis; and aggrecan (*ACAN)*, collagen type II (*COL2b)* and SRY-box 9 (*SOX9*) for chondrogenesis. All samples were run in triplicate using *18S* as a reference gene. Data were quantified using ΔΔCt comparisons.

**Table 2:** Equine primer and probe sequences used for gene expression analyses.

**Gene**	**Primer and probe sequences**
18S, 18 small ribonucleic acid	Forward, 5′- GCCGCTAGAGGTGAAATTCT-3′
	Reverse, 5′- TCGGAACTACGACGGTATCT−3′
	Probe, 5′- AAGACGGACCAGAGCGAAAGCAT-3′
RUNX2, runt-related transcription factor 2	Forward, 5′-GAACCCAGAAGGCACAGACA-3′
	Reverse, 5′-GGCTCAGGTAGGAGGGGTAA-3′
	Probe, 5′-ATTTAGGGCGCATTCCTCATCCCA-3′
ALP, alkaline phosphatase	Forward, 5′-CGGGACTGGTACTCGGAC-3′
	Reverse, 5′-CATGTACTTCCGGCCGCC-3′
	Probe, 5′-AAGGACATTGCCTACCAGCTCGTG-3′
OC, osteocalcin	Forward, 5′-GTGCAGAGTCTGGCAGAGGT-3′
	Reverse, 5′-CCAGCCAATGATCCAGGTAG-3′
	Probe, 5′-AATCTCTTCACCACCTCACTGCCC-3′
PPARγ, peroxisome proliferator-activated receptor	Forward, 5′-TCTCATTGACCCAGAAAGCGA−3'
	Reverse, 5′-CCACTTTGATCGCACTTTGGTA-3′
	Probe, 5′-TGCAAGAGCTGATCCCATGGTTGA-3′
ACAN, aggrecan	Forward, 5′-GAGGAGATGGAGGGTGAGGT−3′
	Reverse, 5′-GATGGTGATGTCCTCCTCGC-3′
	Probe, 5′-TTCACCTGTGTAGCAGATGGCGTC-3′
COL2b, type II collagen	Forward, 5′-GCTACACTCAAGTCCCTCAAC-3′
	Reverse, 5′-ATCCAGTAGTCTCCGCTCTT-3′
	Probe, 5′-ACCTGAAACTCTGCCACCCTGAAT-3′
SOX9, SRY-box 9	Forward, 5′-CTGGAGACTGCTGAACGAGA-3′
	Reverse, 5′-GAGATGTGTGTCTGCTCCGT−3′
	Probe, 5′-AGAAGGACCACCCGGACTACAAGTA-3′

### Biochemical Analyses

SM-MSC and BM-MSC pellets were collected and stored at −20°C in medium prior to biochemical assays. The dimethylmethylene blue (DMMB) spectrophotometric assay (Sigma-Aldrich, St. Louis, MO) was used to quantify proteoglycan content in the pellets after digestion in 0.5 mg/mL papain (Sigma Aldrich St. Louis, MO). Chondroitin-4 sulfate (Sigma-Aldrich, St. Louis, MO) was used to establish a standard curve and the optical density determined at 525 nm (31).

### Statistical Analysis

All data were analyzed using JMP14 (SAS, Cary, NC). A mixed effects model was used to analyze all continuous data including cell proliferation and doubling time, cell surface marker expression, fold change gene expression, and GAG content. Cell proliferation, doubling time, cell surface marker expression and GAG content are expressed as the mean ± SEM. Relative expression is expressed using a box and whisker plot with median (line), upper and lower quartiles (box), and 5% and 95% percentiles (whiskers). Horse was considered as a random effect. Significance was set at *p* < 0.05.

## Results

### Proliferation

**Figure 1:**
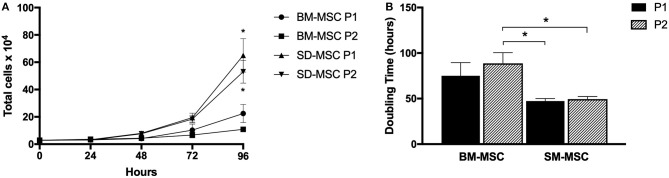
**(A)** Graph showing cell proliferation (mean ± SEM) of P1 and P2 SM-MSCs and BM-MSCs over 96 h. **(B)** Graph showing doubling time (mean ± SEM) of P1 and P2 SM-MSCs and BM-MSCs cultured for a total of 96 h. *p < 0.05.

Cell proliferation and doubling time for P1 and P2 SM-MSCs and BM-MSCs are shown in Figure 1. Both cell types did not demonstrate significant increases in cell numbers until 72 h of culture. At 96 h, both P1 and P2 SM-MSCs had significantly more cells than P1 and P2 BM-MSCs. Doubling time of P1 and P2 SM-MSCs was significantly < P2 BM-MSCs.

### Immunophenotyping

**Figure 2:**
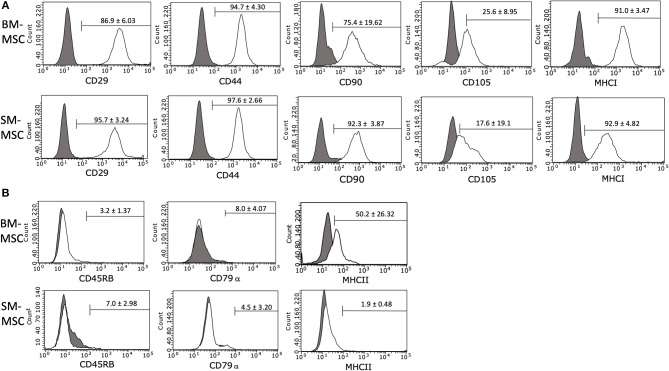
Flow cytometric histogram analyses of cell surface marker expressions in P2 **(A)** BM-MSCs and **(B)** SM-MSCs. The gray lines represent isotype controls and white lines represent respective cell surface marker staining. The mean ± SEM percentage of positive cells is in the corner of each histogram. Each histogram is a representative result of 6 horses.

The immunophenotype of P2 SM-MSCs and BM-MSCs was characterized using flow cytometric analysis of phenotypic cell surface markers including CD29, CD44, CD45RB, CD79α, CD90, CD105, MHCI, and MHCII. SM-MSCs and BM-MSCs exhibited similar immunophenotypic characteristics and both cell types expressed cell surface markers considered consistent with stemness, while simultaneously lacking expression of cell surface markers considered inconsistent with MSCs (Figure 2). Both cell types were strongly positive for expression of CD29, CD44, CD90, and MHCI. Expression of CD105 was moderate in both cell types, 17.6 ± 19.1% of SM-MSCs and 25.6 ± 8.95% of BM-MSCs. SM-MSCs and BM-MSCs were negative for expression of the hematopoietic cell surface markers CD45RB and CD79α. Interestingly, BM-MSCs had moderate expression of the exclusion marker, MHCII, with 50.2 ± 26.32% of cells expressing MHCII. Significant inter-horse variability was also noted. In comparison, only 1.9 ± 0.48% of SM-MSCs expressed MHCII.

Now reads: Flow cytometric histogram analyses of cell surface marker expressions in P2 a) BM-MSCs and b) SM-MSCs.

### Chondrogenic Differentiation Potential

**Figure 3:**
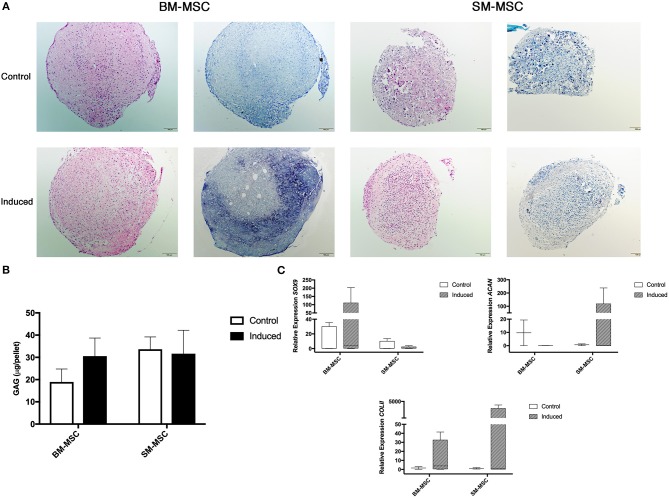
Chondrogenic differentiation of BM-MSCs and SM-MSCs was compared using histology, GAG content of pellets and expression of chondrogenic-related genes. **(A)** Photomicrographs of control and induced BM-MSC and SM-MSC pellets on day 28 stained with H&E and toluidine blue (scale bar = 100μm). **(B)** GAG content in control and induced BM-MSC and SM-MSC pellets on day 28. **(C)** Box plots of expression of chondrogenic-related genes including *SOX9, ACAN*, and *COL2b* for BM-MSC and SM-MSC pellets relative to control BM-MSCs. *18S* was used as a reference gene in all analyses.

Chondrogenic differentiation potential of SM-MSCs and BM-MSCs was compared by assessing MSC pellets cultured over a 28-day period. Grossly, BM-MSC pellets were larger and rounder than SM-MSC pellets (Figure 3A). Histologically, BM-MSC pellets cultured in chondrogenic media exhibited more intense toluidine blue staining consistent with proteoglycan deposition than SM-MSC pellets. Glycosaminoglycan content was not significantly different between any of the treatment groups, although BM-MSC control pellets had the lowest GAG content per pellet (Figure 3B). Overall, expression of markers of chondrogenesis including *SOX9, ACAN*, and *COL2b* displayed significant variability as noted in (Figure 3C). Expression of *SOX9* was increased in induced BM-MSCs compared to control BM-MSCs, however, *SOX9* expression was low in both control and induced SM-MSCs. Expression of *ACAN* and *COL2b* was highest in induced SM-MSCs, although this did not reach statistical significance.

### Osteogenic Differentiation Potential

**Figure 4:**
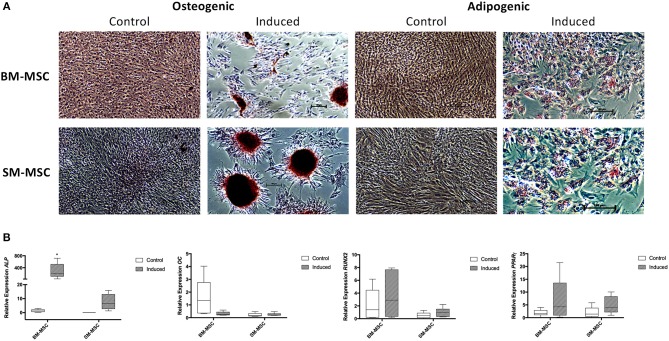
Comparison of osteogenic and adipogenic differentiation of BM-MSCs and SM-MSCs. **(A)** Photomicrographs of control and induced BM-MSCs and SM-MSCs stained with alizarin red (osteogenesis) and oil red O (adipogenesis). Osteogenically induced MSCs stained with alizarin red are positive for extracellular calcium consistent with osteogenesis. Adipogenically induced MSCs demonstrated positive oil red O staining of lipid droplets consistent with adipogenesis. **(B)** Box plots of expression of osteogenic-related genes including *ALP, OC*, and *RUNX2* for BM-MSC and SM-MSC pellets relative to control BM-MSCs and expression of the adipogenic-related gene *PPAR*γ*. 18S* was used as a reference gene in all analyses. *p < 0.05.

Both SM-MSCs and BM-MSCs demonstrated osteogenic differentiation following 14 days of culture in osteogenic media. Alizarin red staining was used to assess presence of calcium, with both cell types demonstrating positive staining compared to control cells (Figure 4A). Control cultures of SM-MSCs and BM-MSCs cultured in basal medium did not show any evidence of histologic differentiation. Expression of *ALP* was significantly increased in induced BM-MSCs compared to control BM-MSCs, control SM-MSCs and induced SM-MSCs (*p* = 0.01). Although expression of *ALP* was increased in induced SM-MSCs compared to control SM-MSCs, this difference did not reach statistical significance. The expression of *OC* was not significantly different between any of the treatment groups. The expression of *RUNX2* was increased in induced BM-MSCs compared to control BM-MSCs and in induced SM-MSCs compared to control SM-MSCs, however, no statistically significant differences were noted (Figure 4B).

### Adipogenic Differentiation Potential

Adipogenic differentiation was observed in both SM-MSCs and BM-MSCs. At 14 days following adipogenic induction, cells demonstrated lipid droplet deposition by positive staining with oil red O (Figure 4A). Control SM-MSCs and BM-MSCs that were not cultured in adipogenic media did not show evidence of adipogenic differentiation histologically. *PPAR*γ expression was increased in induced BM-MSCs compared to control BM-MSCs and in induced SM-MSCs compared to control SM-MSCs, although the differences between groups did not reach statistical significance (Figure 4B).

## Discussion

The main objective of this study was to directly compare the immunophenotype, proliferative potential and chondrogenic capabilities of equine SM-MSCs and BM-MSCs differentiated in pellet culture. Currently, a direct comparison between equine SM-MSCs and BM-MSCs has not been reported. First, we demonstrated that MSCs derived from the synovial membrane and bone marrow of horses have a similar immunophenotype as assessed by cell surface marker expression. However, only BM-MSCs exhibited moderate expression of the exclusion marker, MHCII. We also found that SM-MSCs have superior proliferative capacity and doubling time, when compared to BM-MSCs, during culture expansion. Finally, although moderate chondrogenic differentiation was noted in both BM-MSCs and SM-MSCs, ECM synthesis appeared to be superior in BM-MSCs, while expression of chondrogenic-related genes was increased in SM-MSCs compared to BM-MSCs.

Flow cytometric analysis of cells demonstrated similar immunophenotypes between SM-MSCs and BM-MSCs. Both populations of cells were largely positive for the cell surface markers consistent with stemness including CD29, CD44, and CD90 and negative for exclusion markers including CD45, CD79α, and MHCII. Interestingly, expression of MHCII was consistently low in SM-MSCs (<2%), however, BM-MSCs had moderate expression of MHCII (~50%) with significant variability among different horses. Although MHCII is generally considered to be an exclusion marker for stem cells in most other species, other investigators have reported expression of MHCII in equine BM-MSCs (32). Schnabel et al. found that equine BM-MSCs were heterogenous in their expression of MHCII with variability within individual horses at different bone marrow aspiration times, between different horses, and at different passage numbers. It is possible that BM-MSCs are more susceptible to MHCII expression in comparison to SM-MSCs. This could have important clinical implications as MHCII expression in MSCs has been associated with increased immunogenicity due to allorecognition (32). MHCII expression should likely be minimized when using MSCs clinically in order to prevent cell rejection.

Similar to some other equine studies describing immunophenotyping of SM-MSCs and BM-MSCs, expression of CD105 was somewhat low in this study (23, 33). Although CD105 is considered a marker of stemness in many species (34), CD105 expression appears to be variable in the horse with some studies reporting high expression and others reporting low expression (33, 35). Variability amongst studies may be due to the use of different antibodies as reliable equine-specific antibodies are notoriously difficult to produce. It may also reflect the significant heterogeneity within horse populations compared to syngeneic small animal model populations. Expression of CD105 may be an important variable in differentiation; Harvanova et al. reported that positive selection of CD105^+^ cells from synovial fluid and synovial membrane led to superior differentiation capabilities compared to CD105^−^ cells (36). Because we found that both SM-MSCs and BM-MSCs exhibited only moderate chondrogenic differentiation, selection of CD105^+^ cells could be considered in the future to enhance differentiation as this has not been described in equine MSCs.

Similar to human SM-MSCs, the proliferative capabilities of SM-MSCs appeared to be superior to BM-MSCs (22). Proliferation of P1 and P2 SM-MSCs was significantly higher than BM-MSCs at 96 h and the doubling time of SM-MSCs was significantly lower compared to BM-MSCs. Although a superior rate of cell proliferation is not necessarily required of MSCs, it is important to determine the proliferation rates of MSCs from novel sources in order to assess their suitability for clinical use. This study demonstrates that isolation and culture expansion of equine SM-MSCs from harvested synovial membrane is both practical and feasible.

Successful chondrogenic differentiation of SM-MSCs from other species has been demonstrated including humans and rabbits (37, 38). However, few studies have evaluated chondrogenesis of equine SM-MSCs (18, 23, 26) and a direct comparison of the chondrogenic potential of equine SM-MSCs and BM-MSCs has not been described. We sought to determine if SM-MSCs had superior chondrogenic potential when compared to BM-MSCs due to the need for an improved cell source for cartilage repair. In this study, we found that BM-MSCs showed evidence of superior ECM synthesis when evaluated histologically as pellets demonstrated more intense toluidine blue staining. In contrast to this, we found that SM-MSCs had higher expression of *ACAN* and *COL2b* than BM-MSCs, although statistically significant differences were not detected likely due to large variability in gene expression. It is well known that changes in gene expression are not always reflected in protein synthesis, which may explain the different findings in BM-MSCs and SM-MSCs (39).

The variability in gene expression from pellet cultures is important to address and may be associated with the relative difficulty of extracting RNA from MSC pellets. At 28 days of culture, pellets have synthesized a robust ECM which requires mechanical disruption (biopulverization) prior to RNA extraction. Precise biopulverization in liquid nitrogen was required to ensure that cells were available for lysis. This additional step increases the risk of RNA degradation compared to lysis of cultured cells.

Overall, equine SM-MSCs do not appear to have superior chondrogenic potential when compared to BM-MSCs. Recently, Ogata et al. (22) demonstrated superior chondrogenesis in human SM-MSCs compared to BM-MSCs with SM-MSC pellets being larger, having more intense toluidine blue staining and having increased expression of cartilage specific genes including aggrecan, type II collagen and *SOX9*. However, the MSCs used in this study were pre-sorted such that only LNGFR^+^THY-1^+^ cells were used based on previous work demonstrating that these markers allowed for highly enriched stem cell populations (22, 40). Enhanced chondrogenesis in CD105^+^ cells described by Harvanová et al. (36) and in LNGFR^+^THY-1^+^ cells by Ogata et al. (22) indicate that presorting cells to isolate the desired progenitor population may be needed. This may be especially pertinent in equine MSC populations in which significant cell variability is noted (29). Overall we found only moderate chondrogenesis of both SM-MSCs and BM-MSCs, however, the *in vitro* nature of the study may contribute to diminished chondrogenic potential of MSCs. *In vivo* transplantation of SM-MSCs into cartilage defects has demonstrated effective cartilage repair at 3 months in a pig model and 6 months in a rabbit model, while ectopic chondrogenic differentiation was unsuccessful, indicating that the local environment may play a significant role in cell differentiation (41, 42). Further investigation of chondrogenesis and cartilage repair using equine SM-MSCs *in vivo* is likely warranted.

Both SM-MSCs and BM-MSCs showed evidence of osteogenic and adipogenic differentiation as evidenced by histology and gene expression profiles. BM-MSCs had significantly higher expression of *ALP* compared to SM-MSCs. The expression of *RUNX2* was also higher in BM-MSCs compared to SM-MSCs, although this was not statistically significant. Similar results were described in human BM-MSCs when compared to SM-MSCs (22), therefore, equine BM-MSCs may preferentially differentiate to bone compared to SM-MSCs. Adipogenic differentiation was similar between equine BM-MSCs and SM-MSCs.

It appears that considerable variability exists between species' responsiveness to chondrogenesis. Although SM-MSCs appear to have superior chondrogenic capabilities in other species, in this study under the described culture conditions and with direct comparison to BM-MSCs, we found that equine SM-MSCs were not chondrogenically superior. Repair of chondral defects using MSCs remains challenging, largely due to the need for improved cell-based chondrogenesis. Synovial membrane-derived MSCs have shown superior chondrogenesis in other species, however, no direct comparison with BM-MSCs exists in the horse. Here, we directly compare the immunophenotype, proliferation rate, and chondrogenic differentiation capabilities of equine BM-MSCs and SM-MSCs. Although synovial membrane is a practical and feasible source of MSCs in the horse, superior chondrogenesis *in vitro* should not be expected under currently described culture conditions.

## Data Availability

No datasets were generated or analyzed for this study.

## Ethics Statement

This study was carried out in accordance with the recommendations of the Institutional Animal Care and Use Committee (IACUC) at the University of Pennsylvania.

## Author Contributions

AG contributed to study design, acquisition, analysis and interpretation of data, and preparation of the final manuscript. RL, RM, and GM contributed to acquisition of data. KO contributed to study design, acquisition, analysis and interpretation of data, and preparation of the manuscript. All authors approved the final manuscript.

### Conflict of Interest Statement

The authors declare that the research was conducted in the absence of any commercial or financial relationships that could be construed as a potential conflict of interest.
